# Prevalence and factors associated with the burden of caregivers of older adults with post‐COVID syndrome

**DOI:** 10.1002/alz.090005

**Published:** 2025-01-09

**Authors:** Fabiana Carla Matos da Cunha Cintra, Marco Túlio Cintra, Estevão Alves Vallle, Vitória Lanza Lommez, Júlia Monção Dias Carvalho, Antônio Augusto De Carvalho Duarte, Gerson Santos Azevedo, Iza de Faria Fortini, Bernardo De Mattos Viana, Maria Aparecida Camargos Bicalho

**Affiliations:** ^1^ Federal University of Minas Gerais, Belo Horizonte, Minas Gerais Brazil; ^2^ Clínica Mais 60, Belo Horizonte, Minas Gerais Brazil; ^3^ UFMG, Belo Horizonte Brazil; ^4^ INCT – NeuroTecR and CTMM, Belo Horizonte, MG Brazil; ^5^ Older Adult’s Psychiatry and Psychology Extension Program I Federal University of Minas Gerais, Belo Horizonte, MG Brazil; ^6^ Universidade Federal de Minas Gerais, Belo Horizonte Brazil; ^7^ Department of Mental Health, School of Medicine, Federal University of Minas Gerais, Belo Horizonte, MG Brazil; ^8^ INCT – NeuroTecR and CTMM, Belo Horizonte, Minas Gerais Brazil; ^9^ Graduate Program in Applied Sciences to Adult Health, Faculdade de Medicina, Universidade Federal de Minas Gerais, Belo Horizonte Brazil; ^10^ National Institute of Science and Technology Neurotec R (INCT‐MM), Faculdade de Medicina, Universidade Federal de Minas Gerais, Belo Horizonte Brazil; ^11^ Department of Clinical Medicine, Faculdade de Medicina, Universidade Federal de Minas Gerais, Belo Horizonte Brazil

## Abstract

**Background:**

Post‐Covid syndrome is related to functional, cognitive, psychological and physical impairments in older adults, which has been associated with caregiver burden.

**Method:**

Cross‐sectional study nested within a prospective cohort, which recruited participants aged 60 and over, and their respective caregivers, twelve months after laboratory diagnosis of Covid‐19. The project was approved by UFMG’s ethics committee. The Zarit Burden Interview and Hamilton depression scales were used to assess caregiver burden and depression, respectively. d. Caregiver burden was categorized as present with a cutoff of 15 points. The Chi‐Square and Mann Whitney tests were used for categorical and continuous variables, respectively. Variables with a p value <0.2 were subjected to multivariate analysis using the backward method. The SPSS statistical package, version 25.0, was used.

**Result:**

52 participants and their respective caregivers were evaluated. Among the participants, 60.8% were hospitalized, had a mean age of 72.39±9.1 years, education of 7.67±4.7 years and 51% were female. Caregivers had a mean age of 55.27±15.9 years, education of 10.9±4.8 years, 76.5% were female, 92.1% were spouses or children, 64% lived with the participant and 44% had income between 1 and 2 minimum wages. The prevalence of caregiver burden was 54.2%, being 42.3% of these cases of moderate intensity. An association was observed, in univariate analysis, with the caregiver’s depressive symptoms OR = 1.331 (95%CI 1.109‐1.598; p‐value<0.001), participant’s depressive symptoms OR = 5.429 (95%CI 1.285‐22.941; p‐value = 0.005) and living with the elderly OR = 3.073 (95%CI 1.299‐7.269; p‐value = 0.004). After multivariate analysis, only the caregiver’s depressive symptoms showed an association OR = 1.331 (95% CI 1.109‐1.598; p‐value = 0.002).

**Conclusion:**

The prevalence of burden among caregivers of elderly people with Post‐Covid Syndrome was high and was associated with caregiver’s depressive symptoms, indicating the need for long‐term support and assessment of this population.

